# The relationship between mindfulness, depression, anxiety, and quality of life in individuals with schizophrenia spectrum disorders

**DOI:** 10.1192/j.eurpsy.2021.2079

**Published:** 2021-08-13

**Authors:** N. Bergmann, E. Hahn, I.M. Hahne, M. Zierhut, T.M.T. Ta, M. Bajbouj, M. Pijnenborg, K. Böge

**Affiliations:** 1 Department Of Psychiatry, Charité – Universitätsmedizin Berlin, Campus Benjamin Franklin, Berlin, Germany; 2 Faculty Of Behavioral And Social Sciences, University of Groningen, Groningen, Netherlands

**Keywords:** Depression, Anxiety, mindfulness, Schizophrenia spectrum disorders

## Abstract

**Introduction:**

Mindfulness-based interventions have received growing attention over the last years for the treatment of various mental disorders, including schizophrenia spectrum disorders (SSD), demonstrating their transdiagnostic validity. However, no study has examined the relationship of probable mechanisms underlying the therapeutic effects of mindfulness in SSD.

**Objectives:**

The current study examines the relationship between mindfulness, depression, anxiety, and quality of life in individuals with SSD through quantitative measures.

**Methods:**

A total of 83 participants with SSD were recruited at the in- and outpatient facility of the Charité – Universitätsmedizin Berlin in Germany. Participants completed the Southampton Mindfulness Questionnaire, Comprehensive Inventory for Mindful Experiences, and Freiburger Mindfulness Inventory, the Depression, Anxiety, Stress Scale, and the World Health Organization Quality of Life Questionnaire. PROCESS analysis examined the relationship between mindfulness and quality of life and the mediating role of depression and anxiety.

**Results:**

Indicated a significant positive association between mindfulness and physical health, psychological and environmental quality of life. Depression and anxiety were found to mediate this relationship, with higher depression and anxiety scores being related to lower mindfulness and quality of life. In this relationship, however, depression was found to be the stronger predictor.

**Conclusions:**

The findings of this study provide insight into the mechanisms of mindfulness. Initial evidence for the transdiagnostic and process-based clinical relevance of MBIs for SSD has been found and future studies can further explore the role of mindfulness for central therapeutic processes of change by employing longitudinal designs.

**Disclosure:**

No significant relationships.

